# Supplementary material for: Identifying the topographic signature of early Martian oceans
Source: Nature. 2026 Apr 15;652(8111):30. doi: 10.1038/s41586-026-10381-2 (PMC13102716; doi:10.1038/s41586-026-10381-2)
Supplement: Supplementary file 1 — This file contains Supplementary Figs. 1–4, Supplementary Tables 1 and 2, Supplementary Videos 1 and 2, and additional references. [file 41586_2026_10381_MOESM1_ESM.docx]

Supplementary Information

**Identifying the topographic signature of early Martian oceans**

**Abdallah S. Zaki^1,2^, Michael P. Lamb^1^**

^1^Division of Geological and Planetary Sciences, California Institute of Technology, Pasadena, California, 91125, USA

^2^Department of Earth and Planetary Sciences, Jackson School of Geosciences, The University of Texas at Austin, Austin, TX, USA

Corresponding author. Abdallah.zaki@jsg.utexas.edu

***In this Supplementary Information document, we:***

1. Present a flowchart outlining the workflow used to identify the shelf;
2. Show that slope and elevation patterns remain consistent across different resolutions;
3. Provide a classification of the deltaic deposits used in the analysis;
4. Present trial-and-error results from the Geomorphons terrain classification using different maximum terrain angles;
5. Provide legends for Movies S1 and S2.


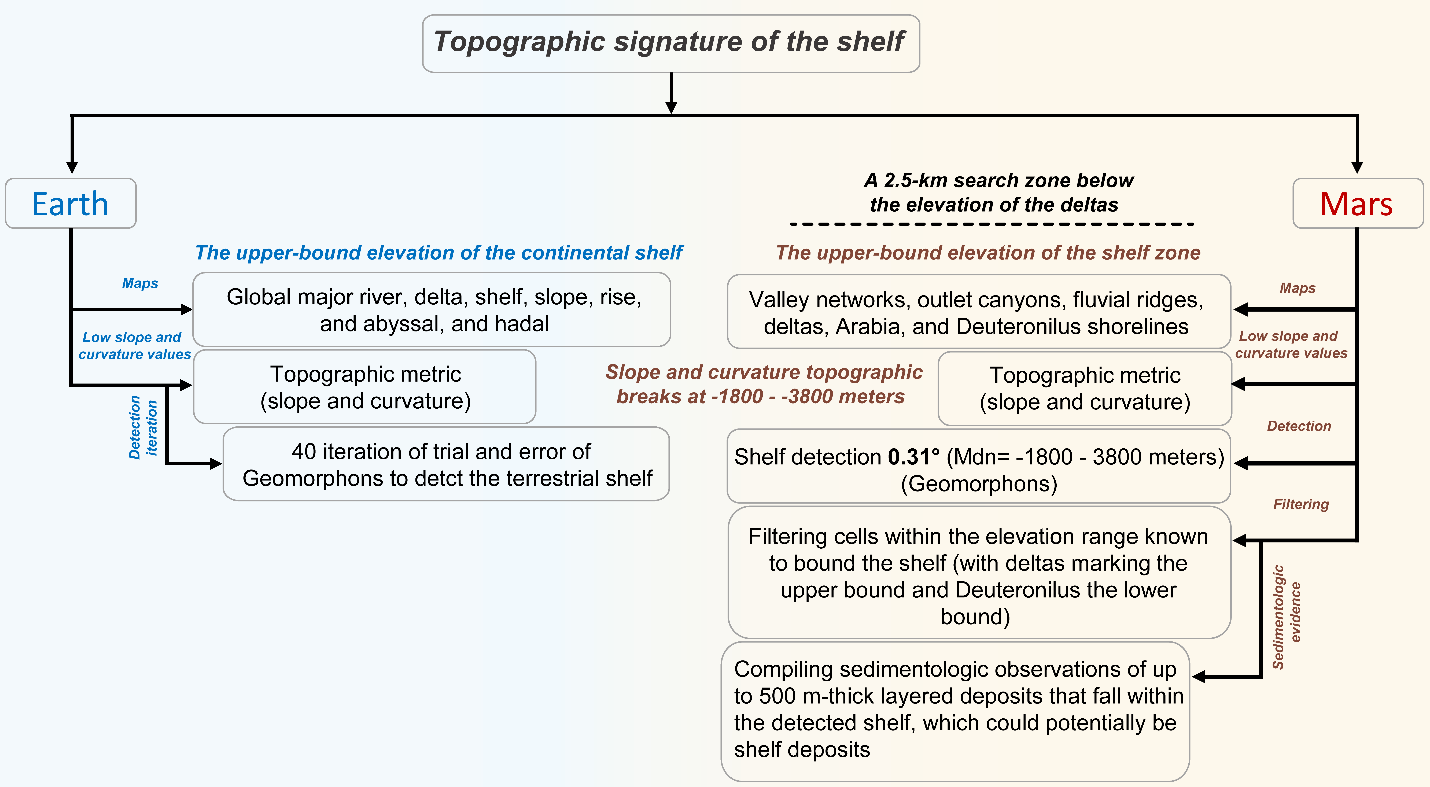


**Fig. S1 | Conceptual workflow for identifying the topographic signature of the shelf on Earth and Mars.** The flowchart outlines the approach used to define and detect shelf-like topography. On Earth, we first used terrestrial landforms (major rivers and deltas) to establish the upper bound of the shelf, followed by mapping the extent of oceanic landforms (continental shelf, slope, rise, abyssal plain, and hadal zone). We then described these landforms topographically to characterize the shelf and applied this understanding to detect and refine its expression. For Mars, we followed a similar approach using valley networks, outlet canyons, fluvial ridges, deltas, and the Arabia and Deuteronilus margins to set the upper bound of the search zone. We then analyzed the surface topography within the first 2.5 km below the delta elevations to determine whether distinct topographic metrics were present. A median slope value of 0.31°—characteristic of the –1,800 m to –3,800 m zone—was used to identify cells with similar topographic signatures. These cells were filtered to fall between the known delta elevations and the Deuteronilus boundary. Finally, we compiled sedimentologic observations of layered deposits that could potentially represent preserved shelf deposits.


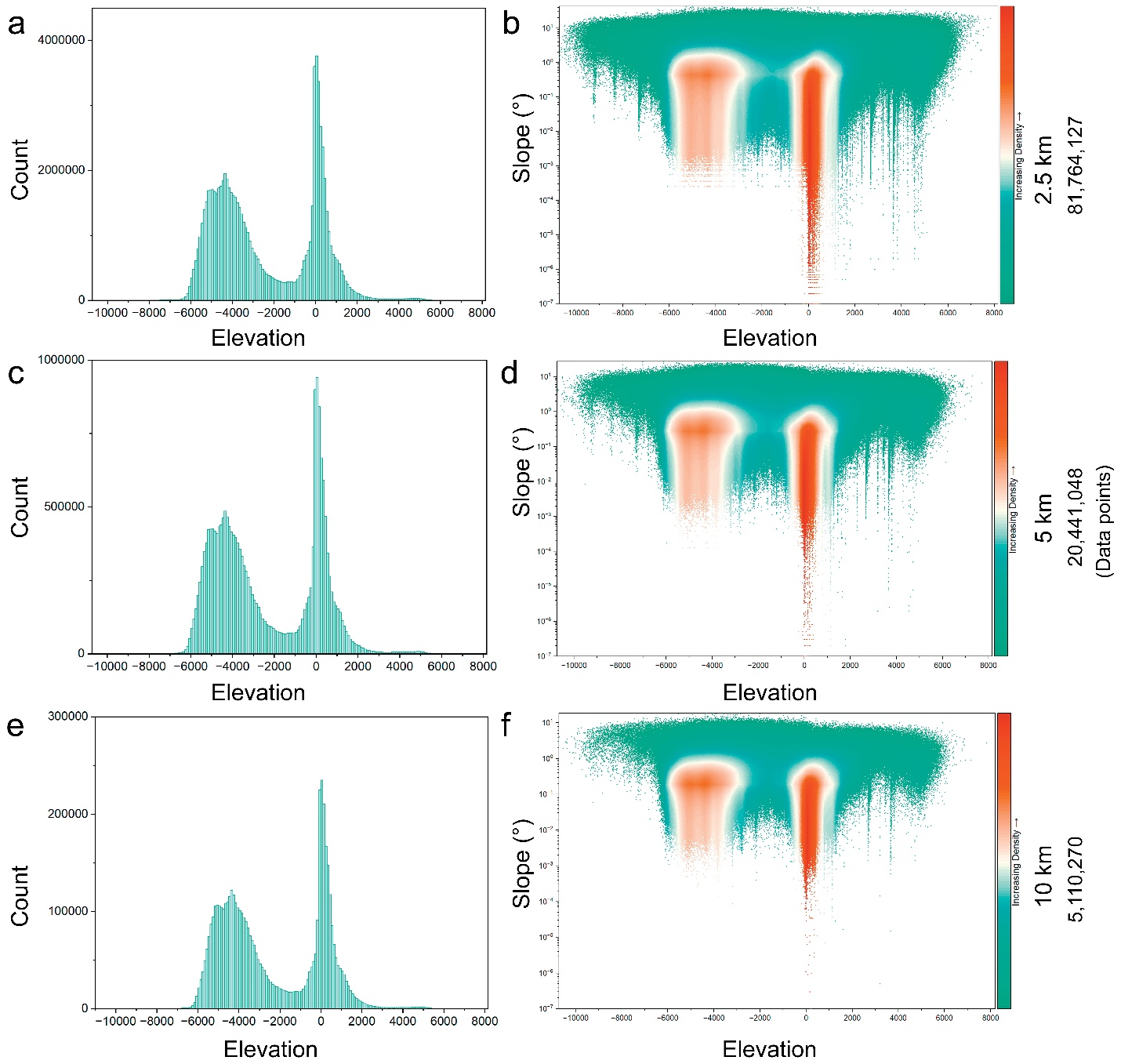


**Fig. S2 | Slope and elevation values for Earth at different pixel resolutions (2.5 km, 5 km, and 10 km per pixel).** The slope and curvature data were computed from the ETOPO Global Relief Model using ArcGIS (see Methods). The figure demonstrates that while the specific values of slope and elevation change with pixel resolution, the overall spatial pattern remains consistent.


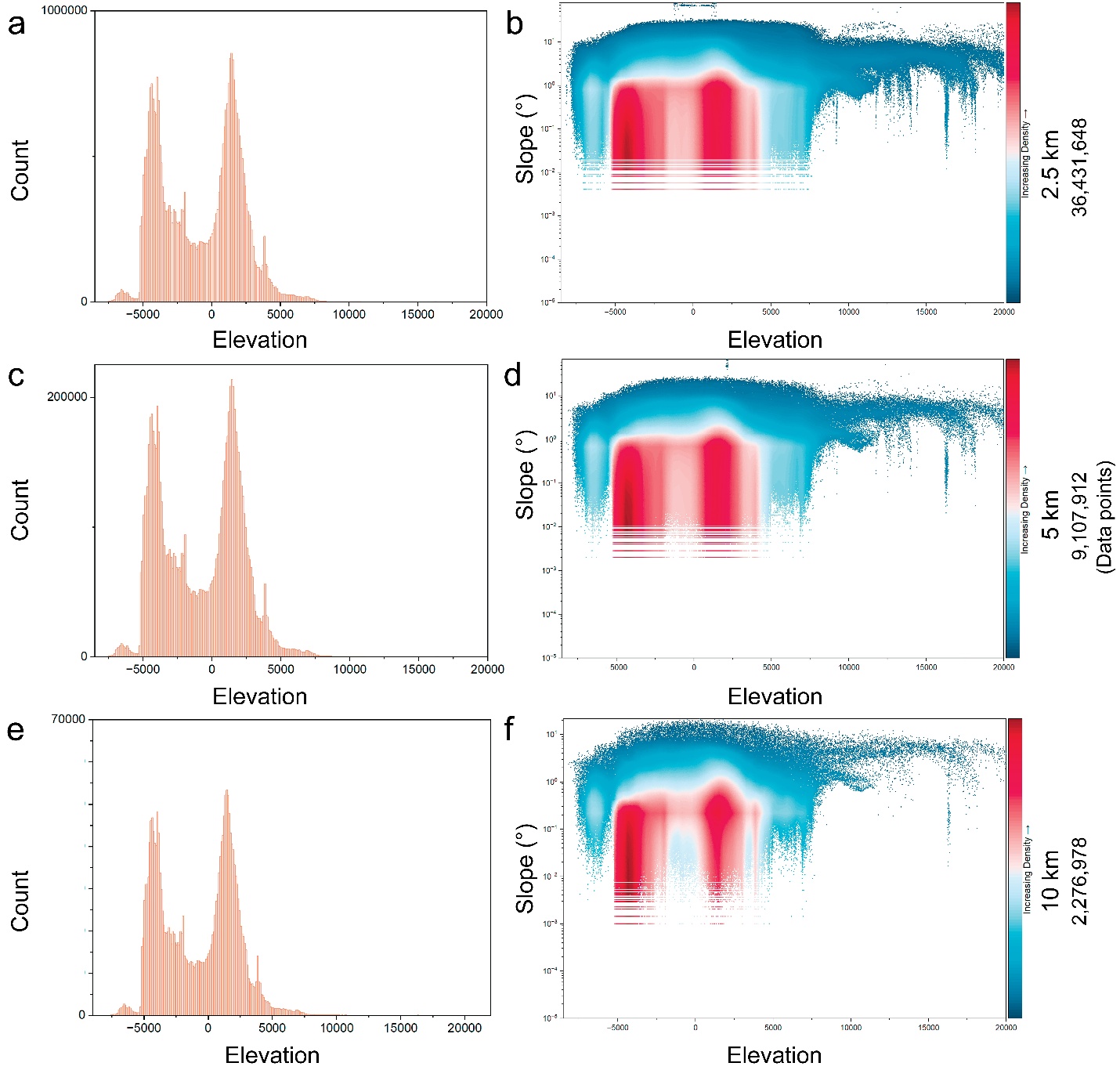


**Fig. S3 | Slope and elevation data for Mars at various pixel resolutions (2.5 km, 5 km, and 10 km per pixel).** These values were calculated using ArcGIS and the global Mars Orbiter Laser Altimeter (MOLA) gridded topography (see Methods). The figure illustrates that although slope and elevation values vary with resolution, the overall spatial distribution remains similar across scales.


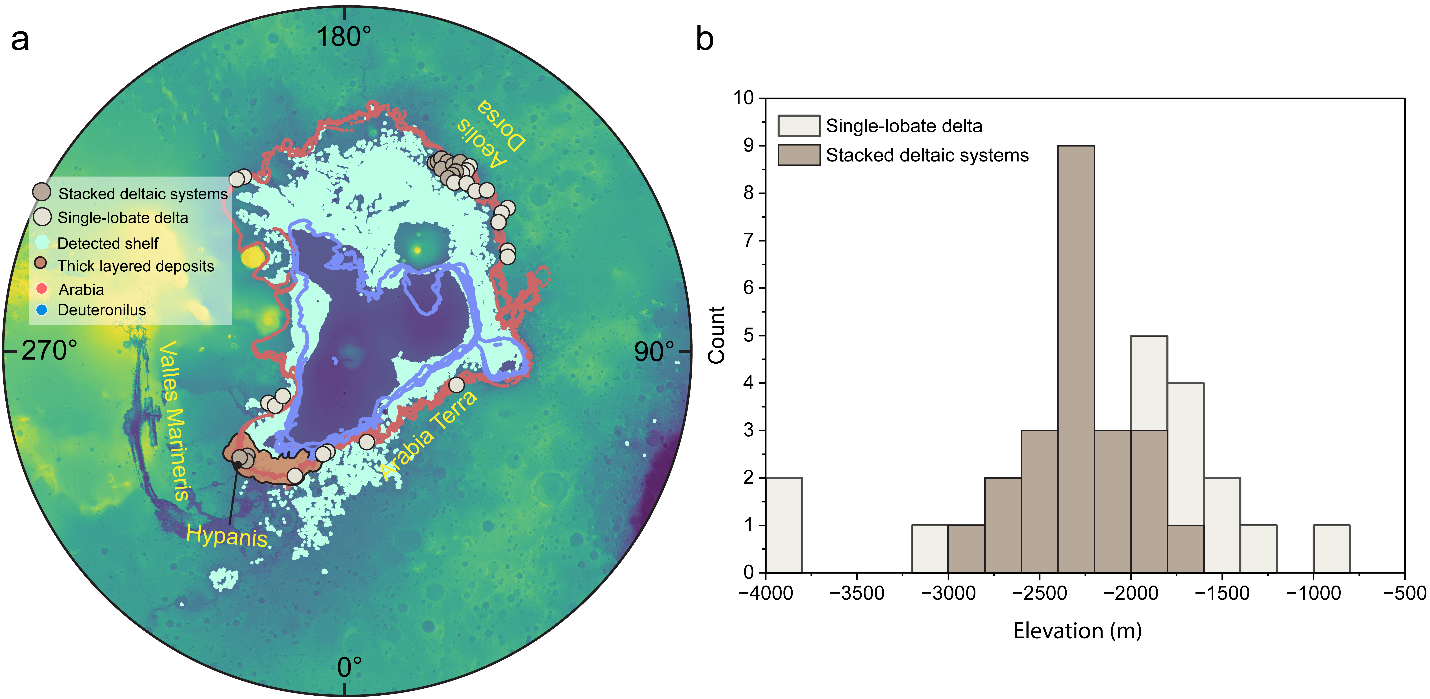


**Fig. S4 | Interpreted deltas and deltaic-to–submarine-channel successions along the proposed shorelines, detected shelf cells, and layered deposits. a, Global distribution of deltas—both open-basin and closed-basin—examined in this study, shown together with the proposed shorelines, detected shelf cells, and thick layered deposits. Background map is based on MOLA topography. The dataset was compiled and filtered based on previous mapping in refs.1–8 (Table S1). b, Histogram showing the elevation ranges of the two delta categories.**

**Table S1.** **The examined open-basin deltaic features along the dichotomy boundary.** This dataset was compiled from the following studies 1-8. Systems are classified based on their spatial and sedimentologic context as either single-lobate deltas or stacked deltaic systems. Stacked systems comprise large deltaic successions that have previously been identified and interpreted as recording shoreline regression at Hypanis and shoreline transgression at Aeolis Dorsa^3–5^, whereas isolated systems along the dichotomy boundary are classified here as single-lobate deltas. In some cases, deltaic systems in Aeolis Dorsa are connected to channels that have been interpreted to represent submarine channels.

| **Longitude** | **Latitude** | **Elevation (m)** | **Classification** |
| --- | --- | --- | --- |
| 150.3957 | -2.57724 | -2467 | Stacked deltaic systems |
| 151.5326 | -5.34104 | -2491 | Stacked deltaic systems |
| 150.9137 | -4.96192 | -2405 | Stacked deltaic systems |
| 150.558 | -4.69387 | -2293 | Stacked deltaic systems |
| 149.1995 | -2.7041 | -2268 | Stacked deltaic systems |
| 150.0536 | -3.35775 | -2391 | Stacked deltaic systems |
| 153.7412 | -4.08644 | -2108 | Stacked deltaic systems |
| 154.6857 | -4.59259 | -2299 | Stacked deltaic systems |
| 154.909 | -4.98344 | -2341 | Stacked deltaic systems |
| 149.3736 | -2.52552 | -2400 | Stacked deltaic systems |
| 155.0388 | -4.69236 | -2245 | Stacked deltaic systems |
| 154.8564 | -4.53841 | -2218 | Stacked deltaic systems |
| 153.679 | -6.01441 | -2323 | Stacked deltaic systems |
| 154.5498 | -6.66616 | -2137 | Stacked deltaic systems |
| 153.1269 | -6.59133 | -2075 | Stacked deltaic systems |
| 149.1735 | -6.28996 | -1982 | Stacked deltaic systems |
| 150.8376 | -6.85914 | -1983 | Stacked deltaic systems |
| 149.3147 | -5.96477 | -1802 | Stacked deltaic systems |
| 148.7687 | -5.74833 | -1706 | Stacked deltaic systems |
| -45.4158 | 11.35919 | -2604 | Stacked deltaic systems |
| -44.5966 | 11.58354 | -2665 | Stacked deltaic systems |
| -44.9182 | 13.10371 | -2880 | Stacked deltaic systems |
| -147.21 | -5 | -2531 | Single-lobate deltas |
| 148.47 | -7.75 | -1586 | Single-lobate deltas |
| 148.7 | -7.2 | -1993 | Single-lobate deltas |
| 148.29 | -7.44 | -1806 | Single-lobate deltas |
| -23.8943 | 17.83914 | -2978 | Single-lobate deltas |
| 149.76 | -7.71 | -1699 | Single-lobate deltas |
| 149.94 | -7.93 | -1756 | Single-lobate deltas |
| -15.168 | 31.603 | -3941 | Single-lobate deltas |
| 132.832 | -5.059 | -2359 | Single-lobate deltas |
| 132.694 | -3.615 | -2237 | Single-lobate deltas |
| 131.193 | -1.654 | -1815 | Single-lobate deltas |
| 121.636 | 2.167 | -1916 | Single-lobate deltas |
| 147.827 | -7.462 | -1638 | Single-lobate deltas |
| 140.492 | -5.63 | -2270 | Single-lobate deltas |
| 142.634 | -3.725 | -2452 | Single-lobate deltas |
| 144.791 | -4.05 | -2211 | Single-lobate deltas |
| 122.409 | 1.704 | -933 | Single-lobate deltas |
| -52.881 | 36.517 | -3193 | Single-lobate deltas |
| -55.944 | 44.194 | -2203 | Single-lobate deltas |
| 9.7 | 37.603 | -2709 | Single-lobate deltas |
| -146.354 | -5.28035 | -2200 | Single-lobate deltas |
| 71.944 | 29.462 | -1702 | Single-lobate deltas |
| 148.003 | -7.625 | -1592 | Single-lobate deltas |
| 147.411 | -6.649 | -1827 | Single-lobate deltas |
| -14.404 | 32.19 | -3823 | Single-lobate deltas |
| -57.254 | 34.931 | -1240 | Single-lobate deltas |

**Table S2.** Trial and error using Geomorphons terrain classification for different maximum terrain angles. Results show the terrain area (at ~1.6 x ~1.6 km grid cell resolution) that was correctly and incorrectly identified as continental shelf, as defined by previous mapping (*9*).

| **Run No.** | **Maximum terrain angle ( ͦ )** | **Detected area rea at specific terrain angle (km^2^)** | **Total area of detected cells that correspond to the mapped shelf (km^2^)** | **Percent of areas correctly identified** | **Area of detected cells that do not correspond to the mapped shelf (km^2^)** | **Percent of areas that do not correspond to the shelf** | **Areas of the shelf that were not detected (km^2^)** | **Areas of the shelf that were not detected** |
| --- | --- | --- | --- | --- | --- | --- | --- | --- |
| 1 | 0.01 | 953693 | 477853 | 1.5 | 475840 | 49.9 | 31764692 | 98.5 |
| 2 | 0.02 | 3141193 | 1655086 | 5.1 | 1486107 | 47.3 | 30587459 | 94.9 |
| 3 | 0.03 | 6204915 | 3187174 | 9.9 | 3017741 | 48.6 | 29055371 | 90.1 |
| 4 | 0.04 | 9603489 | 4806557 | 14.9 | 4796931 | 49.9 | 27435988 | 85.1 |
| 5 | 0.05 | 12925097 | 6288288 | 19.5 | 6636809 | 51.3 | 25954257 | 80.5 |
| 6 | 0.06 | 16193424 | 7660595 | 23.8 | 8532829 | 52.7 | 24581950 | 76.2 |
| 7 | 0.07 | 19398629 | 8909908 | 27.6 | 10488721 | 54.1 | 23332637 | 72.4 |
| 8 | 0.08 | 22586358 | 10063033 | 31.2 | 12523325 | 55.4 | 22179512 | 68.8 |
| 9 | 0.09 | 25741303 | 11111961 | 34.5 | 14629342 | 56.8 | 21130584 | 65.5 |
| 10 | 0.1 | 28866211 | 12072680 | 37.4 | 16793531 | 58.2 | 20169865 | 62.6 |
| 11 | 0.11 | 31979441 | 12960441 | 40.2 | 19019000 | 59.5 | 19282104 | 59.8 |
| 12 | 0.12 | 35078577 | 13779876 | 42.7 | 21298702 | 60.7 | 18462669 | 57.3 |
| 13 | 0.13 | 38163289 | 14532814 | 45.1 | 23630475 | 61.9 | 17709731 | 54.9 |
| 14 | 0.14 | 41232690 | 15233392 | 47.2 | 25999299 | 63.1 | 17009153 | 52.8 |
| 15 | 0.15 | 44304544 | 15891198 | 49.3 | 28413346 | 64.1 | 16351347 | 50.7 |
| 16 | 0.16 | 47369985 | 16508641 | 51.2 | 30861344 | 65.1 | 15733904 | 48.8 |
| 17 | 0.17 | 50430628 | 17091810 | 53.0 | 33338818 | 66.1 | 15150735 | 47.0 |
| 18 | 0.18 | 53474889 | 17641712 | 54.7 | 35833177 | 67.0 | 14600833 | 45.3 |
| 19 | 0.19 | 56515464 | 18162593 | 56.3 | 38352871 | 67.9 | 14079952 | 43.7 |
| 20 | 0.2 | 59524231 | 18650898 | 57.8 | 40873334 | 68.7 | 13591647 | 42.2 |
| 21 | 0.21 | 62535445 | 19118544 | 59.3 | 43416901 | 69.4 | 13124001 | 40.7 |
| 22 | 0.22 | 65532194 | 19564504 | 60.7 | 45967690 | 70.1 | 12678041 | 39.3 |
| 23 | 0.23 | 68505830 | 19987388 | 62.0 | 48518442 | 70.8 | 12255157 | 38.0 |
| 24 | 0.24 | 71475981 | 20394120 | 63.3 | 51081862 | 71.5 | 11848425 | 36.7 |
| 25 | 0.25 | 74414043 | 20780235 | 64.4 | 53633808 | 72.1 | 11462310 | 35.6 |
| 26 | 0.26 | 77341749 | 21149241 | 65.6 | 56192507 | 72.7 | 11093304 | 34.4 |
| 27 | 0.27 | 80245679 | 21500839 | 66.7 | 58744840 | 73.2 | 10741706 | 33.3 |
| 28 | 0.28 | 83131205 | 21838059 | 67.7 | 61293145 | 73.7 | 10404486 | 32.3 |
| 29 | 0.29 | 85991447 | 22161227 | 68.7 | 63830220 | 74.2 | 10081318 | 31.3 |
| 30 | 0.3 | 88836570 | 22473059 | 69.7 | 66363511 | 74.7 | 9769486 | 30.3 |
| 31 | 0.31 | 91649737 | 22771879 | 70.6 | 68877858 | 75.2 | 9470666 | 29.4 |
| 32 | 0.33 | 97210900 | 23332144 | 72.4 | 73878783 | 76.0 | 8910401 | 27.6 |
| 33 | 0.35 | 102691427 | 23852540 | 74.0 | 78838887 | 76.8 | 8390005 | 26.0 |
| 34 | 0.4 | 115990743 | 24999725 | 77.5 | 90991018 | 78.4 | 7242820 | 22.5 |
| 35 | 0.6 | 163671127 | 28127836 | 87.2 | 135543291 | 82.8 | 4114709 | 12.8 |
| 36 | 0.8 | 203990151 | 30013010 | 93.1 | 173977141 | 85.3 | 2229535 | 6.9 |
| 37 | 1 | 238151117 | 31279267 | 97.0 | 206871850 | 86.9 | 963278 | 3.0 |
| 38 | 1.2 | 267078221 | 32191561 | 99.8 | 234886660 | 87.9 | 50984 | 0.2 |
| 39 | 1.21 | 268406667 | 32231259 | 100.0 | 236175408 | 88.0 | 11286 | 0.0 |
| 40 | 1.22 | 269721337 | 32270206 | 100.1 | 237451131 | 88.0 | -27661 | -0.1 |

**Movie S1 | Sea-level transgression of ~900 m based on deltaic deposits at Aeolis Dorsa, Mars.** This animation illustrates sea-level change over time, inferred from interpreted deltaic deposits and submarine channel belts at Aeolis Dorsa^5^. These deposits indicate a sea-level rise from approximately −2475 m to −1693 m.

**Movie S2 | Sea-level regression of 500 m based on deltaic deposits at Hypanis Valles, Mars.** This animation shows a sea-level drop of nearly 500 m^3^, from approximately −2500 m to −3000 m.

**Supplementary references**

1. Di Achille, G. & Hynek, B. M. Ancient ocean on Mars supported by global distribution of deltas and valleys. *Nat. Geosci.* **3**, 459–463 (2010).
2. Sholes, S. F. & Rivera-Hernández, F. Constraints on the uncertainty, timing, and magnitude of potential Mars oceans from topographic deformation models. *Icarus* **378**, 114934 (2022).
3. Fawdon, P. *et al.* The Hypanis Valles Delta: The last highstand of a sea on early Mars? *Earth Planet. Sci. Lett.* **500**, 225–241 (2018).
4. Hughes, C. M., Cardenas, B. T., Goudge, T. A. & Mohrig, D. Deltaic deposits indicative of a paleo-coastline at Aeolis Dorsa, Mars. *Icarus* **317**, 442–453 (2019).
5. Cardenas, B. T. & Lamb, M. P. Paleogeographic reconstructions of an ocean margin on Mars based on deltaic sedimentology at Aeolis Dorsa. *J. Geophys. Res. Planets* **127** (2022).
6. Quantin-Nataf, C. et al. Oxia Planum: The landing site for the ExoMars “Rosalind Franklin” rover mission: Geological context and prelanding interpretation. Astrobiology 21, 345–366 (2021).
7. Rivera‐Hernández, F. & Palucis, M. C. Do deltas along the crustal dichotomy boundary of Mars in the gale crater region record a northern ocean? *Geophysical Research Letters* **46,** 8689–8699 (2019).
8. De Toffoli, B., Plesa, A.-C., Hauber, E. & Breuer, D. *Delta deposits on Mars: A global perspective* (2021). doi:10.5194/egusphere-egu21-5971
9. Harris, P. T., Macmillan-Lawler, M., Rupp, J. & Baker, E. K. Geomorphology of the oceans. *Marine Geology* **352**, 4–24 (2014).
